# Adaptive mechanisms to drought risk management in a KwaZulu-Natal community, South Africa

**DOI:** 10.4102/jamba.v17i1.1757

**Published:** 2025-04-15

**Authors:** Vuyiswa Khumalo, Hloniphani Moyo, Lutendo Mugwedi, Johanes Belle

**Affiliations:** 1 Disaster Management Training and Education Centre, Faculty of Natural and Agricultural Sciences, University of the Free State, Bloemfontein, South Africa; 2 Department of Geography and Environmental Sciences, Faculty of Science, Engineering and Agriculture, University of Venda, Thohoyandou, South Africa

**Keywords:** climate change, adaptive mechanisms, human outcomes, livelihoods, resilience, survival, sustainability

## Abstract

Households in developing countries have established various informal mechanisms to cope with unanticipated disasters such as droughts. Understanding these strategies in the context of climate change will enhance the design of locally applicable adaptation mechanisms. This study aimed to investigate a South African rural community’s adaptive mechanisms *during*- and *ex-post*-drought. Questionnaires were administered to 200 household heads, and five focus group discussions were held with selected community members. An index was computed to establish each adaptive mechanism’s importance (termed the rank order) relative to its use in the study area. *During* droughts, borrowing from neighbours was the most commonly used mechanism by households with no savings, while sharecropping was the second most used mechanism. *Ex-post*-droughts, using savings was the primary strategy for community members who had savings, followed by seeking employment as the second most used strategy.

**Contribution:**

Findings from the study suggest that the community is highly vulnerable to droughts and disaster risks because of poor adaptive mechanisms, overreliance on water-dependent activities and lack of adequate support from different stakeholders. Community members use different mechanisms *during*- and *ex-post*-drought, depending on the stage or severity of the drought. The government and stakeholders should promote community awareness and early warning systems for droughts to mitigate disaster risks. These initiatives should ideally be combined with strengthening existing response measures and educating communities to adequately prepare for droughts and their aftermath.

## Introduction

Climate change is expected to increase the severity, duration and frequency of extreme events such as floods and droughts, posing significant disaster risks that threaten livelihoods and food security for millions of poor people (Kim & Jehanzaib 2020). The impacts of droughts affect economies worldwide and may linger for years after the disaster has passed (Liu & Zhou 2021). In southern Africa, droughts will become more severe and frequent, heightening disaster risks, increasing food insecurity and causing a loss of livelihood options (Orimoloye et al. 2022). African populations, particularly those mostly living under the international poverty line and those in the semi-arid subtropics have been suggested to be vulnerable to droughts, caused mainly by unfavourable weather patterns, long dry seasons and climatic variations (Ngcamu & Chari 2020). Within South Africa, provinces such as the Eastern Cape (Muyambo, Jordaan & Bahta 2017) and Limpopo (Nembilwi et al. 2021) have also been mentioned as severely affected by past droughts, and as being vulnerable to future droughts. Vulnerable and impoverished communities are likely to continue experiencing the impacts of these disasters because of their high dependence on natural resources to make a living, lack of financial support and low capacity to adapt to a drop in resource availability (Pamla, Thondhlana & Ruwanza 2021).

A community’s vulnerability determines its susceptibility to droughts, establishing a relationship between vulnerability, disaster risk and adaptive capacity (Mugandani et al. 2022). For vulnerable communities, their adaptive capacities are regarded as their ability to adjust to severe conditions such as droughts, moderate potential damages and cope with the consequences (Sander-Regier et al. 2009). Adjusting, moderating and coping with drought effects include adopting irrigation techniques and introducing drought-tolerant crops (Ruwanza, Thondhlana & Falayi 2022). Therefore, a less vulnerable community, that is, one with access to early warning information, diversified income sources and access to water (Ncube, Mangwaya & Ogundeji 2018), has a more robust adaptive capacity in the event of a drought, and as vulnerability increases, disaster risk and adaptive capacity decline (Shikwambana, Malaza & Ncube 2022). Thus, a lack of adaptive capacity means the community is at greater risk of being exposed to adverse drought effects. Several factors increase a community’s vulnerability and risk to disasters, such as lack of awareness about droughts, lack of early warning systems, poverty and weak institutions (Lottering, Mafongoya & Lottering 2021).

Previously, drought management mechanisms in South Africa relied on reactive, short-term response approaches of providing post-drought relief and introducing restrictions on water supply (Ruwanza et al. 2022). However, Birkmann et al. (2022) revealed that introducing vulnerability and impact assessments improves understanding of who is most at risk and the different approaches that may be implemented to reduce the harsh impacts of such disasters. The current study was designed to investigate the causes of vulnerability and disaster risk for the community in Abaqulusi Local Municipality (ALM 2021) towards drought, emphasising establishing their adaptation and coping mechanisms following the most recent drought (2014–2016) and other drought events in the past.

This study uses the adaptive capacity framework to analyse the ability of the community to adjust to the challenges brought about by droughts (Sarker et al. 2020). The framework has been developed to have a better understanding of vulnerability as a function of people’s exposure and adaptive capabilities to environmental change and risks to disasters (e.g. Sander-Regier et al. 2009). The community’s adaptive capacity through its social, economic and institutional support was viewed as pivotal in reducing vulnerability and disaster risk associated with drought (Figure 1-A1). Because climate change is expected to exacerbate existing climate-related risks, such as droughts and other disasters, communities are anticipated to implement strategies that enhance their resilience to future-related risks. As such, resilience through establishing robust adaptive mechanisms should be viewed as a combination of adaptive, transformative and absorptive capacities aimed at reducing vulnerability (see Sarker et al. 2020, for a more in-depth discussion). Therefore, understanding a community’s vulnerabilities, adaptation and adaptive mechanisms to drought will enhance government stakeholders’ efforts in mitigating disaster risks and improve the community’s own effectiveness when intervening in future drought events.

### Contextual background

South Africa has a long history of drought events, and during the 2014–2016 drought, KwaZulu-Natal Province was declared a disaster area because of the high risk it faced (Strydom & Salvage 2017). Within the Province, ALM was one of the municipalities hit the hardest by the drought, and some communities still face the drought’s adverse effects, with severe social, economic and environmental impacts (ALM Annual Report 2016). During and after the drought (2014–2016), these impacts were severe at both community and household levels, as there were job losses and increases in food prices, contributing to high poverty and child malnutrition levels (Ruwanza et al. 2022).

## Research methods and design

### Description of the study area

The study area is located in the northern part of the KwaZulu-Natal Province in South Africa and is one of the five local municipalities under the Zululand District Municipality (Figure 1). The area is occupied by approximately 211 060 people, with a geographical coverage estimated at 4185 km^2^ in extent, making it one of the spatially largest municipalities in the province (Abaqulusi IDP 2021). The study area comprises rural and urban settlements, with Vryheid being its main urban settlement (Ntshangase 2014). The ALM has a viable economic structure, which relies on farming, mining, timber and small business industries (Abaqulusi IDP 2021). The area is subject to summer rainfall with dry winters, with rain predominantly falling in summer, from November to March (Abaqulusi IDP 2021). The mean annual precipitation ranges between 493 mm – 700 mm, while daily temperatures in summer range from 19°C to 32°C and 3.5°C to 17°C in winter (Ntshangase 2014).

**FIGURE 1:**
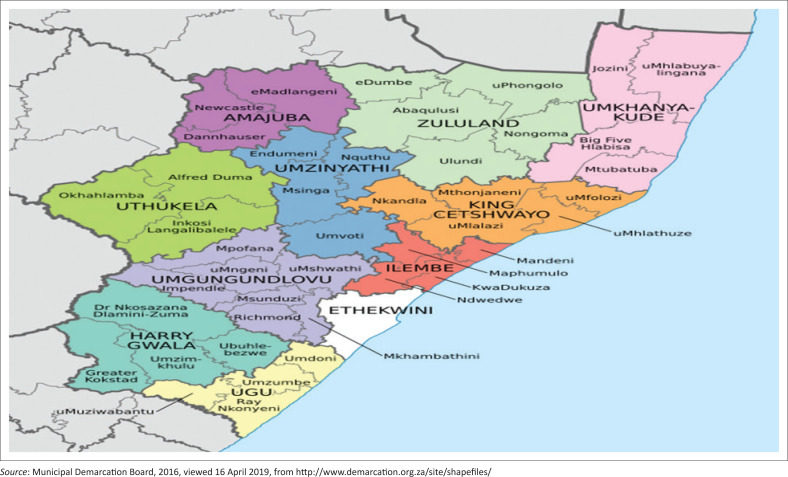
Map showing the location of the Amajuba and Abaqulusi Local Municipality in KwaZulu-Natal’s Zululand District Municipality, South Africa.

### Sampling procedure

The sampling frame for the study consisted of randomly selecting 10 wards from the total 22 wards within ALM. Population numbers differ within the 22 wards, with some wards consisting of over 10 000 inhabitants, while other wards have under 2000 people (Abaqulusi IDP 2021). Only six wards had populations less than 2000, nine wards had between 2000 and 10 000 and seven wards had populations exceeding 10 000. The two extremes in terms of population distribution (wards with the least [less than 2000] and those with the most people [exceeding 10 000]) were selected for sampling. Within each ward, 20 households were selected using a simple random sampling technique, totalling 200 households for the study. Five focus group discussions (FGDs) were also held to extract more information from a separate representative population sample.

### Data collection methods

#### Household questionnaire

Approximately 200 households were surveyed using interviewer-administered questionnaires. As much as the situation permitted, the household head (HH) was targeted to represent each household. When the selected HH was unavailable or unwilling to participate, a different household was considered for sampling. For the household questionnaire, closed-ended questions were used, and the questionnaire was guided by the following two key questions:

- What adaptive mechanisms have you employed to reduce the impacts and disaster risks of drought, *during*- and *ex-post-drought*?
- What assistance was offered by the government and other stakeholders to reduce the impacts and disaster risks *during*- and *ex-post-drought*?

The during- or ex-post-drought period was defined as:

- *During* – for a period of between 9 and 12 months from the declaration of the drought period.
- *Ex-post* – for a period of at least 12 months post the drought period.

#### Focus group discussions

Five FGDs were held to assess the different adaptation mechanisms used by households to reduce their vulnerability to drought impacts. Focus group discussion participants differed from those selected to participate in the household questionnaire. Each group had eight participants who were community members, traditional leaders, ward counsellors and committee members. The guiding questions asked during household interviews (described in the section ‘Household questionnaire’) also guided the FGDs although FGD questions were open ended. During FGDs, discussions also focussed on the community’s vulnerability to droughts because open-ended questions permitted respondents to elaborate more and describe their own experiences in more detail. The FGDs were conducted in IsiZulu to allow all members to participate freely, as it is the most spoken language in the area.

### Data analyses

Responses from the questionnaire were coded by assigning a numerical value to each response and only affirmative responses were expressed in percent. The data obtained from FGDs were handled using thematic analysis, producing key themes on understanding participants’ responses by grouping respondents’ answers to each question and identifying common themes and quotations from participants. These themes were the perceptions on common causes of vulnerability, adaptation mechanisms employed by community members, preparedness measures by the government and stakeholders and government interventions and shortcomings to enhance ALM’s adaptive capacity to drought.

From the first stage of piloting the study on 10 households interviewed, at least three adaptive mechanisms were identified as most common for each phase (*during*- and *ex-post-drought*). These included: doing nothing, reducing food consumption and using savings (*during* a drought); looking for a job, relying on charity and migrating to the city (*ex-post*-drought). An adaptation mechanism index (AMI) for individual adaptive mechanisms was computed to determine how many respondents used the adaptation mechanisms (adopted and modified from Mardy et al. 2018). This index established the importance (termed the rank order) of the individual adaptive mechanism relative to its use (Equation 1). This index (termed the adaptation strategy index) was developed for a similar micro-level study in Bangladesh to establish the number of farmer respondents who used drought coping strategies when exposed to the disaster (Mardy et al. 2018). For this current study, the extent of the participants’ practice of different drought adaptive mechanisms was assessed by asking them about the frequency with which they implemented these mechanisms to mitigate disaster risks. The responses obtained were scaled as frequently, occasionally, rarely and not at all. The corresponding scores of 3, 2, 1 and 0 were adapted, respectively (Equation 1):

AMI=ASr×3+ASo×2+ASs×1+ASn×0 [Eqn 1]

where: AMI is the adaptation mechanism index, ASr is the number of responses with regular practice of drought adaptive mechanisms, ASo is the number of responses with occasional practice of drought adaptive mechanisms, ASs is the number of responses with rare practice of drought adaptive mechanisms and ASn is the number of responses with no practice of drought adaptive mechanisms. Therefore, the most used mechanism produced the highest AMI value.

### Ethical considerations

Ethical clearance to conduct this study was obtained from the University of the Frees State Ethics Committee (No. UFS-HSD2017/0822).

## Results and discussion

### Socio-economic characteristics of the respondents

#### Gender and age dynamics

Females comprised a slightly higher proportion of the respondents (52%) compared to males (48%). This distribution aligns with findings from the GHS (2020), which reported that 52% of the population in the area were females, while males accounted for 48%. Most respondents were 36–45 years old, with a combined 33% proportion of the total population. In this category, females contributed a slightly higher proportion of the respondents (19%), compared with 14% of males. The age group that participated the least was the 46–55-year group, with slightly more females (5%) than males (3%) (Figure 2-A1). These results fit well in a rural setting where more men migrate to towns or nearby industrial areas in search of jobs to support their families (Selod & Shilpi 2021).

The rural-to-urban push in search of employment opportunities has contributed to the migration of the male population to urban areas, leaving their families in rural areas. Similar results have been reported in Iran (Mianabadi et al. 2022), in India (Karutz & Kabisch 2023) and in Malawi (Becerra-Valbuena & Millock 2021) when drought was identified as the chief reason for male rural-urban migration. Moreover, the traditional belief that a woman’s place is in the kitchen, as mentioned by Nehemia and Lenkoe (2023), may have also contributed to a higher proportion of women who participated in the household questionnaires. This is because most women were found in their homes looking after their children, while their husbands were reported to be away for work. Somewhat similar results were reported in India (Afridi, Mahajan & Sangwan 2022) and in Uganda (Agamile, Dimova & Golan 2021) when more women did not migrate to urban areas in search of opportunities, instead fending for families through various activities such as agriculture and selling of handicrafts.

#### Education level

A small proportion (8%) of respondents did not have any form of schooling, with females contributing slightly more than males (Figure 2-A1). The highest proportion of education among the respondents was recorded under secondary schooling, followed by tertiary education. Surprisingly, females (28% for secondary and 15% for tertiary education) recorded slightly higher proportions than males (26% and 9%, respectively). At least 25% of both males and females who were secondary-schooled reported having completed Grade 12, but because of financial constraints, they could not pursue their studies at the tertiary level. In a society where the gender gap exposes more males than females to education opportunities (Adeleken & Bussin 2022), this study found different results, with females recording higher proportions in secondary and tertiary education. Perhaps the results could be attributed to the shift towards emphasising females going to school in this community or because more females were found during questionnaire administering than males. The level of education has been reported to have a significant positive effect in improving the resilience of rural communities in the face of droughts in Iran (Savari, Damaneh & Damaneh 2023), while low literacy levels had a negative impact on coping and adaptation strategies in South Africa’s Limpopo Province (Shikwambana et al. 2022). Higher education enables individuals to pursue various livelihood strategies, achieve associated goals and have access to and understanding of different technologies (Savari et al. 2023). It also enables access to information as well as improving the probability of accepting change in the form of adopting new technologies. Therefore, participants in the study area can be viewed as having low resilience to droughts because of their low education levels.

#### Employment status

The study’s findings revealed that ALM has a high unemployment rate, as slightly more than half of the participants (53%) indicated that they were not formally employed (Figure 2-A1). More females (37%) than males (16%) were unemployed, while a small proportion of the study population was formally employed (10%). Formal employment was mainly through government as civil servants, while others were employed in the private sector. Only 10% of the participants indicated that they were working in other sectors, such as domestic work, where they did not need to sign any contract but received a monthly income. The rest of the study population (37%) indicated that they were self-employed and made a living through home entrepreneurship (selling groceries and clothes) and farming as a source of livelihood.

Unemployment in South Africa is generally high, with several people who, after obtaining higher-level tertiary postgraduate education, find themselves unemployed (Mseleku 2022). According to Ahmad, Yaseen and Saqib (2022), the ability of households to adapt to drought and mitigate disaster risk largely depends on available financial capital. Therefore, high unemployment implies that community members have limited capital and adaptation alternatives in the event of a disaster. This exacerbates their vulnerability and exposes them to severe drought impacts and disaster risks, such as increasing food prices and malnutrition (Ruwanza et al. 2022).

#### Source of income and living history in the Abaqulusi Local Municipality area

Social grants were the main source of income, with at least 32% of the participants indicating that their families relied on them for survival (Figure 3-A1). Social grants are given once a month to the retired population (> 60 years), as an allowance to disabled people, and to children (child grant) under the age of 18, whose parents are not formally employed (Van der Berg, Patel & Bridgman 2022). According to Van der Berg et al. (2022), almost a third of the country’s population receives social grants in South Africa. Statistics further show that the KwaZulu-Natal Province has more recipients when compared to the other eight provinces in the country. These facts support the notion that the general population relies on the social grant as a safety net, particularly because unemployment is high and other livelihood alternatives have been eroded. The effects of low unemployment further increase the general population’s vulnerability and disaster risk during a drought, as adaptive mechanisms are limited.

### Adaptive mechanisms employed by households *during*-drought

When asked about any adaptive mechanisms related to reducing the impact of droughts, the highest proportion (56%) highlighted that they had adaptive mechanisms, with males contributing slightly more (30%) than females (26%) (Figure 2). Respondents with no adaptive mechanisms were 26%, again with slightly more males than females (Figure 2). These results suggest that as more than half of the respondents had adaptive mechanisms, most people in ALM could be expected to adapt when exposed to future drought events.

**FIGURE 2:**
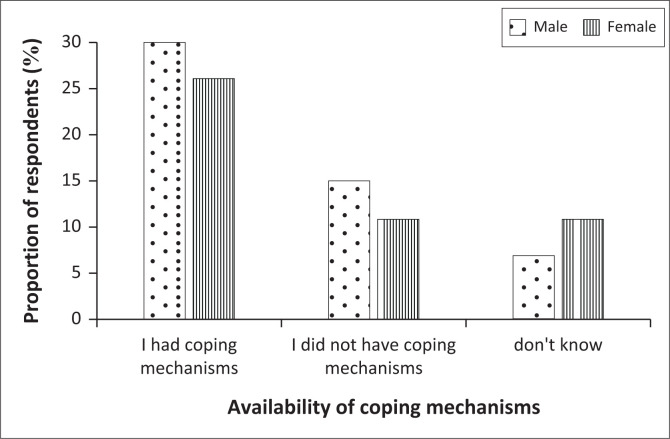
The comparison of respondents who had adaptive mechanisms and those without, to reduce the impact of droughts in the Abaqulusi Local Municipality (ALM), KwaZulu-Natal Province, South Africa.

### Drought adaptive mechanisms: *During* the drought and *ex-post*-drought

***During-drought adaptive mechanisms*:** Several adaptive mechanisms were practised by community members, such as borrowing from neighbours, sharecropping, selling assets and reducing food consumption (Figure 3-A1). To indicate the perceived importance and extent of the use of individual mechanisms, an AMI was computed. Table 1 presents the rank order of various drought adaptive mechanisms reported by farmers in the study area. Borrowing from neighbours emerged as the most important mechanism, with an AMI of 173. Additionally, receiving donations from relatives, borrowing money and obtaining food parcels and other essentials aided community members in adapting to and managing disaster risks during the challenging times of drought. After borrowing, community members repaid neighbours when the drought had passed or when their conditions had improved. Most of these community members mentioned not having substantial savings to fall back on as a first response mechanism; hence borrowing and relying on donations became an easier option. Assistance from family and friends or other relations, to cope with reduced levels of food security, has also been reported by Ansah, Gardebroek and Ihle (2021) in Ghana and Keshavarz, Karami and Vanclay (2013) in rural Iran. Similar results were also reported in Bangladesh (Rabbani & Hasan 2021), Ethiopia (Gebre, Ayenew & Biadgilign 2021) and India (Debnath & Nayak 2022).

**TABLE 1:** The rank order of the adaptive mechanisms utilised during a drought event in Newcastle’s Abaqulusi Local Municipality.

Adaptive mechanisms	Extent of practice				AMI	Rank order
	Frequently	Occasionally	Rarely	Not at all		
Borrowing from neighbours	49	11	4	0	173	1
Share cropping	29	28	4	0	147	2
Use of savings	29	22	2	0	133	3
Crop diversification	25	4	9	0	92	4
Selling of assets	23	6	6	0	87	5
Reducing food consumption	15	17	2	0	81	6
Doing nothing	19	4	3	0	68	7

Note: The AMI represents the adaptive mechanism index calculated to indicate the mechanism most used to adapt to drought.

AMI, adaptation mechanism index.

Code: Frequently = 3, Occasionally = 2, Rarely = 1, Not at all = 0.

For most of the community members, cultivating crops was mentioned as a source of livelihood and sustenance in ALM. Therefore, extended periods of drought impacted food security and livelihood options. As such, sharecropping was the second most important adaptive mechanism (AMI = 147) used *during* an extended drought. Focussing more effort on cultivating crops for survival amid a drought was also reported in Kenya (Quandt 2021), Ethiopia (Gebre et al. 2021) and Uganda (Agamile et al. 2021). In exchange for part of the harvested crop yields, community members highlighted that they offered labour to till land owned by wealthy community members with wells and boreholes for irrigation. This way, their families received food such as maize and dry beans because they did not own boreholes or wells to irrigate their farmlands. However, this mechanism faced challenges as there were water governance and usage limitations or regulations *during*-droughts that limited overuse and over-extraction by those possessing water sources. Short-term labour was also reported as a coping strategy for droughts in Kenya (Quandt 2021) and India (Debnath & Nayak 2022).

As the third most important mechanism (Table 1), community members used their little savings to adapt to droughts and mitigate disaster risks (AMI = 133). This included using cash savings to purchase food, health supplies and particularly water, which was rationed in most parts of ALM *during*-droughts. Similar results, in terms of using cash savings as an important adaptive strategy, were reported by Ncube et al. (2018) in Zimbabwe and by Ansah et al. (2021) in northern Ghana. The fact that using savings was the third most important option suggests that community members were willing to explore other adaptation mechanisms to address the effects of drought and mitigate disaster risks before resorting to their saved money. While some community members surprisingly mentioned doing nothing to adapt to the effects of drought, this strategy was the least important (AMI = 68).

The two most common mechanisms identified across all FGDs were storing water in large containers ahead of time and preserving foodstuff, which included drying meat and fruits and pickling vegetables such as cucumbers and tomatoes during droughts. Household members also stored portions of dry beans and maize (corn) harvests for emergency use, such as *during* a drought:

‘While the 2014–16 drought was severe, my family usually stores excess harvest from our field for emergencies. We managed to draw from the stored maize from my barn, while I also had reserves of dried meat from a previously slaughtered goat. However, these stored resources could only last us for a certain period of time as the drought dragged for longer than we thought, pushing us to resort to other means to survive.’ (Female, 68 years, focus group 2)

In terms of those who did not store water, they had to purchase from local supermarkets at exorbitant costs, while those who relied on government assistance cited waiting on water delivery in tanks as a way of adapting to water shortage. The government also occasionally supplied food parcels as handouts to disadvantaged families, perhaps suggesting that the government was not doing enough. To adapt to the lack of water in the area, community members resorted to using greywater through recycling bathing and kitchen-used water. This water irrigated home vegetable gardens, while mid-sized farmers drastically reduced their farming area or abandoned the fields altogether. Where they maintained smaller planted areas, they watered using buckets instead of flood or drip irrigation. Cultivating crops also shifted to drought-tolerant crops such as sorghum and small areas of maize to avoid crop loss. Such a shift in reducing cropping area size has been reported elsewhere in South Africa’s Limpopo Province and Zimbabwe’s highlands (Masunungure & Shackleton 2018). In our study, large-scale farmers mentioned having little option but to abandon farming land altogether to concentrate on home vegetable gardens. This shift to more manageable vegetable gardens under drought conditions has also been reported in South Africa’s Eastern Cape Province (Andrew & Fox 2004; Shackleton & Luckert 2015).

***Ex-post-drought adaptive mechanisms*:** Community members utilised different mechanisms to adapt to the after-effects of droughts (Table 2, Figure 4-A1). To note, some of these mechanisms were different from the ones used *during* the drought period. The use of savings (AMI = 280) ranked as the most important mechanism *ex-post*-drought, with community members using savings to purchase food and other household essentials. This mechanism ranked as the third most important mechanism used *during*-droughts (see ‘During-drought adaptive mechanism’ section). Interestingly, *ex-post*-drought, this mechanism was not used by people who borrowed from their neighbours *during*-droughts, but rather by those people who used other mechanisms such as sharecropping and crop diversification.

**TABLE 2:** The rank order of the adaptive mechanisms utilised after a drought event in Newcastle’s Abaqulusi Local Municipality.

Adaptive mechanisms	Extent of practice				AMI	Rank order
	Frequently	Occasionally	Rarely	Not at all		
Use savings	43	74	2	0	280	1
Look for a job	43	51	9	0	241	2
Reduce consumption	33	64	3	0	231	3
Borrow from neighbours	23	45	1	0	160	4
Reduce expenditures	19	40	8	0	146	5
Exploit CPRs	13	39	7	0	125	6
Rely on charity	19	24	11	0	116	7
Migrating	15	28	3	0	104	8
Sell off assets	19	6	6	0	76	9

Note: The AMI represents the adaptive mechanism index calculated to indicate the mechanism most used to adapt to the after-effects of a drought.

AMI, adaptation mechanism index; CPR, common property resource.

Code: Frequently = 3, Occasionally = 2, Rarely = 1, Not at all = 0.

As Ellis (2000) discussed, households tend to explore other low-cost options for adapting to disasters and managing risk. For example, households would rather use cash savings when facing a disaster or shock rather than depleting their productive assets. This is also true for our current study because selling assets *ex-post*-drought to adapt was the least important mechanism (AMI = 76). For households with savings, a disaster such as drought increases the likelihood of using their savings first instead of depleting their assets (Doss et al. 2018). Because of the high cost of household essentials and food caused by drought (Devereux 2007), community members in our study reverted to looking for a job (AMI = 241) as a second means of adapting to *ex-post*-droughts. This approach also included possible migration to other areas if there was no employment in the local area (AMI = 104) as the eighth most important mechanism. This generated income through remittances and improved the livelihoods of family members left behind in ALM, a mechanism also reported by Ansah et al. (2021) in northern Ghana.

As a sixth adaptive mechanism, and one that did not appear under the mechanisms used *during*-drought, community members exploited common property resources (AMI = 125). This involved hunting for game in the bush, intensively searching for fruits and nutritious roots and walking long distances to fish. Such a mechanism has been reported in Kenya’s Kitui County (Mosberg & Eriksen 2015) and in Papua, Indonesia (Boissière et al. 2013). However, these practices face significant challenges because of the overuse of common property resources in developing countries (Brossette, Bieling & Penker 2022).

With limited livelihood options *ex-post*-droughts, FGD participants opted for alternative mechanisms such as starting business ventures that included carpentry (wooden chairs and dressers) and welding metal gates/bars to sell. Community members who ventured into these opportunistic and reactive businesses indicated a steady flow of income, although such business ventures increased crime, school dropouts and teenage pregnancies:

‘After a drought, young adults in the community go into the bushes to harvest wood for us to make benches and chairs intended for selling. My son decided to venture into welding big gates and bars for houses. These activities are tricky because crime increases because thieves target our equipment.’ (Male, 54 years, focus group 5)

As a last resort, participants mentioned migrating to the nearest cities in search of livelihood alternatives to adapt to the effects of droughts and mitigate disaster risks. This particularly applied to the area’s youth, with the elderly having limited options but to remain behind.

### Interventions by the local municipality *during* and post-drought

When asked about any potential interventions rendered by ALM during the most recent and other drought events, 40% of the female and 20% of male HH respondents indicated awareness of some form of assistance from ALM (Figure 3a). More males (26%) than females (10%) were unaware that the local municipality aided the community in adapting. Regarding the specific interventions offered by ALM, water provision services ranked high (Figure 3b). More females (38%) than males (8%) mentioned that ALM mainly repaired the non-functioning boreholes to improve water availability for household use.

**FIGURE 3:**
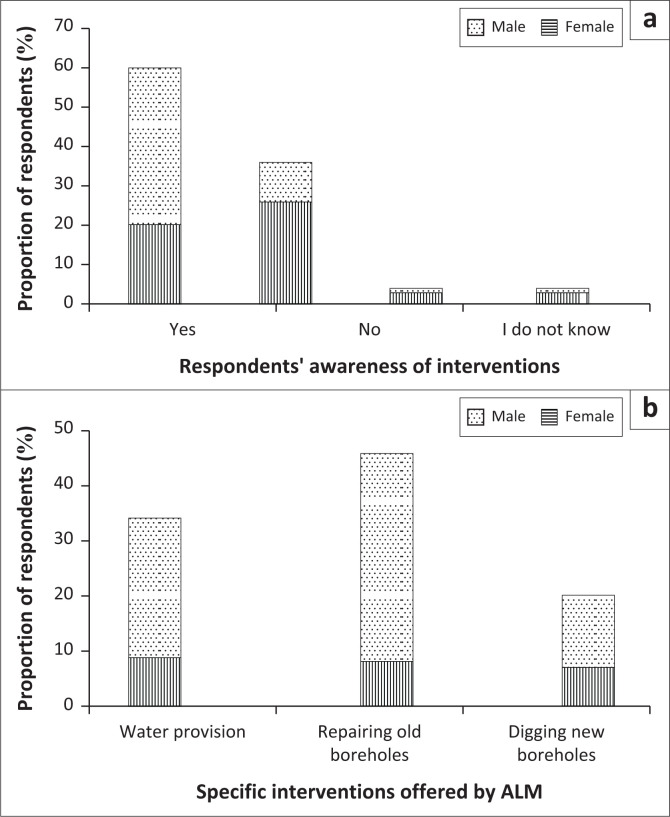
The (a) respondents’ awareness of municipal interventions; and (b) the specific interventions offered by the Abaqulusi Local Municipality (ALM), KwaZulu-Natal Province, South Africa.

Apart from repairing boreholes, ALM also provided supplemental water through delivery tanks at designated points, with a total of 34% of respondents confirming that this intervention was delivered every fortnight. However, most respondents (a combined proportion of 80%) felt that these boreholes did not benefit the whole community, as water demand was more than that supplied from the boreholes. Respondents opined that even though ALM delivered water, its cleanliness was questionable and not everyone could access it, particularly older persons who lived far from where water tankers were stationed. Community members were also concerned about the stipulated water delivery schedule by ALM. Instead of weekly deliveries, it was delivered fortnightly, leading to severe shortages within the community.

While a proportion of 60% reflects considerable involvement by ALM to reduce the impacts of droughts, more could still be implemented to increase interventions that seek to improve the adaptive capacity of community members. South Africa has several policies and legislation that, when implemented, will minimise the impacts of droughts. For example, the Disaster Management Act (DMA) provides a framework for response to natural or human-caused disasters. However, for these legislations to be put into action and be effective, there must be a functional disaster management centre and qualified personnel to carry out disaster management duties:

‘There are several droughts that I have witnessed in the country. However, the area lacks functional structures that translate the interventions from policy material on paper to actual ground implementation for people to adjust and adapt to the effects of droughts and other disasters.’ (Male, 69 years, focus group 4)

### Other stakeholder intervention *during*- and *ex-post*-drought

During the FGDs, respondents indicated that apart from ALM, no stakeholders, governmental agencies or non-governmental organisations were active in reducing the adverse impacts of droughts in the ALM. Besides ALM, other stakeholders identified were: humanitarian organisations, civil society organisations, churches and other government departments. Interventions by stakeholders included: the issuing of food vouchers, provision of water, medical support on malnutrition cases, a supply of animal feed and awareness campaigns about drought and drought-resilient crops:

‘In the past droughts, we have received food vouchers for us to go and exchange at the shops and water tanks. These have mostly been NGOs and some good wealthy samaritans within the society. However, we feel the strategy should be to equip us with more mechanisms that build our capacity for longer-term adaptability and to reduce disaster risk, such as giving us Jojo tanks for us to harvest rainwater and use this during times of poor rains.’ (Female, 55 years, focus group 2)

Although examples of interventions were mentioned (e.g. medical support on malnutrition cases, and a supply of animal feed), their contribution was still reported as low as highlighted during the FGDs. Furthermore, the ineffectiveness of these interventions was caused by stakeholders focussing on selected areas, no consultation with community members before implementing interventions and limited funding, which led to poor coordination of some programmes. The findings of this study are consistent with those of Olaleye (2010), who reported that while some stakeholders were visible during a drought in South Africa’s Free State Province, there were still shortcomings mostly associated with inadequate coordination of programmes and lack of funding, which made the interventions less effective. Therefore, stakeholder intervention is important in reducing the impacts of drought, and allocation of disaster management funding to various government departments can help them attend to drought-related issues in communities.

### The vulnerability of households to drought

The FGDs revealed that households most vulnerable to drought were: poor households with limited or no financial resources to adapt to droughts, households that relied on agricultural production to make a living, pastoralists, women-headed and households with family members employed in the agricultural sector. Also, women and children were especially vulnerable to drought effects because they were left behind while men were away employed in the cities. This feedback from the FGDs is unsurprising given that the agriculture sector relies heavily on rainfall availability, and low rainfall eventually impacts employment and income opportunities. The community relied on seasonal employment on commercial farms: harvesting fruits, vegetables and other produce. Hence, lack of rainfall significantly reduced the probability of employment.

During the FGDs, it was highlighted that most communal farmers were severely affected as they could not plant during the drought, severely impacting farmers’ food security. An increasing level of vulnerability was revealed, particularly among women, and future droughts were suggested to likely cause further negative adjustments in their lifestyles, impacting their already fragile adaptive mechanisms. According to Kim and Jehanzaib (2020), the challenges that women face during droughts include loss of income, increased prevalence of health problems related to the drought such as malnutrition and water-borne diseases (such as cholera) and inability to take care of the educational needs of their children because of limited financial resources:

‘Another drought in the future would be catastrophic and will decimate our already fragile food security levels. During a drought in ALM, women walk long distances to collect water from government water tanks or boreholes, but oftentimes, the water that they collect does not meet the needs of their families. In the absence of men, the women bear the full brunt of a drought event as they need to fend for their families through intensive labor-demanding activities.’ (Male, 73 years, focus group 3)

The FGDs also revealed that most women in ALM rely on community investment societies (stokvels) for savings. However, *during*-drought, most women could no longer continue saving money as their finances were affected and relied on loan sharks to meet their families’ financial obligations. This further increased their vulnerability as paying back loans, with high interests, and seeking alternative livelihood sources, exposed HHs, particularly women-headed households, to high risks of health conditions such as increased blood pressure, anxiety and high stress levels. Therefore, our findings suggest that vulnerability depends on a combination of factors such as income, occupation, family structure, gender and health status of household members, as argued by Hermans and Garbe (2019). To reduce the impacts of droughts, community members, stakeholders and political leaders must work together collaboratively in designing plans that are aimed at addressing the causal factors of vulnerability in the ALM. Immediate action will prevent further drought-related damage and improve social well-being and economic productivity (Kim & Jehanzaib 2020).

## Conclusion

This study sought to understand factors contributing to the vulnerability of people to drought, their adaptive mechanisms *during*- and *ex-post*-drought and the role played by the government and different stakeholders in mitigating the impact of droughts. Interestingly, community members adjusted their adaptive mechanisms *during*- and *ex-post*-droughts, with some mechanisms appearing *ex-post*-droughts, which were otherwise expected to appear *during* the drought period. For example, exploitation of common property resources such as hunting was expected to appear *during*-drought but only appeared *ex-post*-drought. Drysdale, Bob and Moshabela (2021) have also reported such shifting of adaptive approaches in South Africa’s KwaZulu-Natal Province. For the current study, mechanisms such as using savings and reducing food consumption appeared both *during*- and *ex-post*-drought, with savings identified as the most important and used mechanism *ex-post*-drought. Such mechanisms imply that communities adjust and, to a certain extent, adapt according to the timing and, perhaps, the severity of the drought by seeking different alternatives to source livelihood options for sustenance.

By investigating how community members adjust their adaptive mechanisms during and after droughts, this study offers valuable insights to government officials, non-governmental organisations and policymakers in the study area and beyond. It highlights the factors that contribute to people’s vulnerability to disasters such as droughts. As a result, these stakeholders, including civil society organisations, can collaboratively design early warning systems, policies and plans to better equip communities to respond to and adapt to droughts. Such interventions can enhance the adaptive capacity of rural communities. Because drought is a climatic event that cannot be prevented, effective interventions and preparedness will help build more resilient communities to mitigate its impact (Solh & Van Ginkel 2014).

## Figures and Tables

**FIGURE 1-A1:**
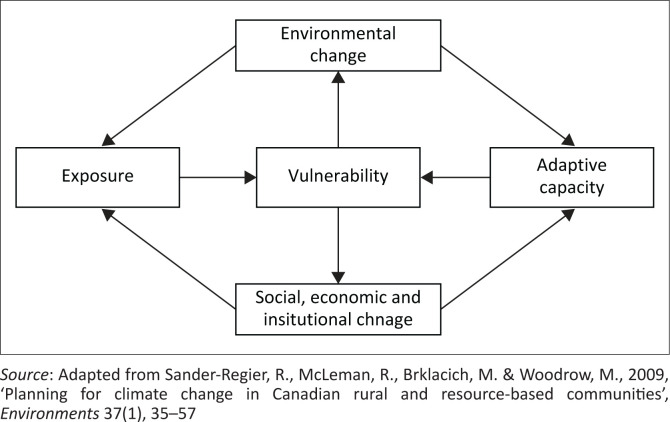
A community’s vulnerability because of its adaptive capacity, exposure to environmental changes, as well as social and institutional changes.

**FIGURE 2-A1:**
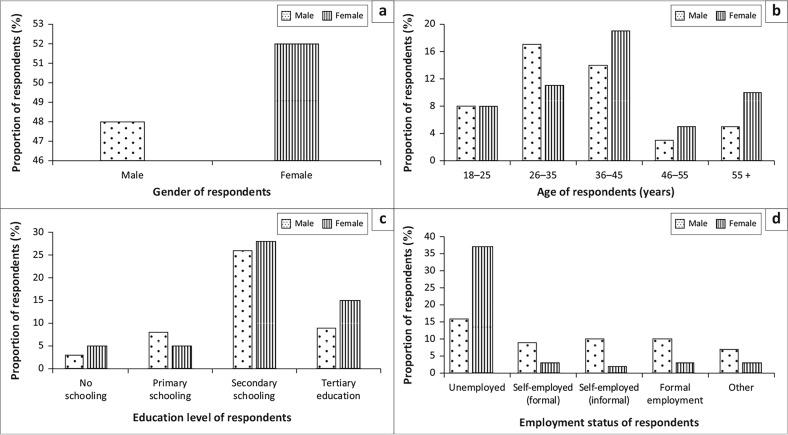
The (a) gender, (b) age, (c) education level and (d) employment status for the household questionnaire survey of respondents in the Abaqulusi Local Municipality (ALM), KwaZulu-Natal Province, South Africa.

**FIGURE 3-A1:**
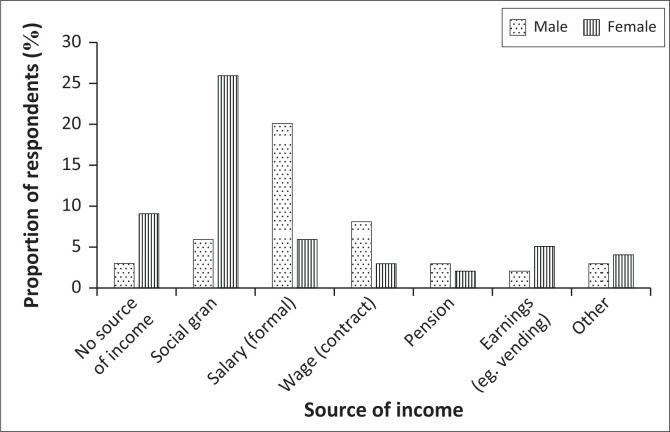
The source of income for the participants in the Abaqulusi Local Municipality (ALM), KwaZulu-Natal Province, South Africa.

**FIGURE 4-A1:**
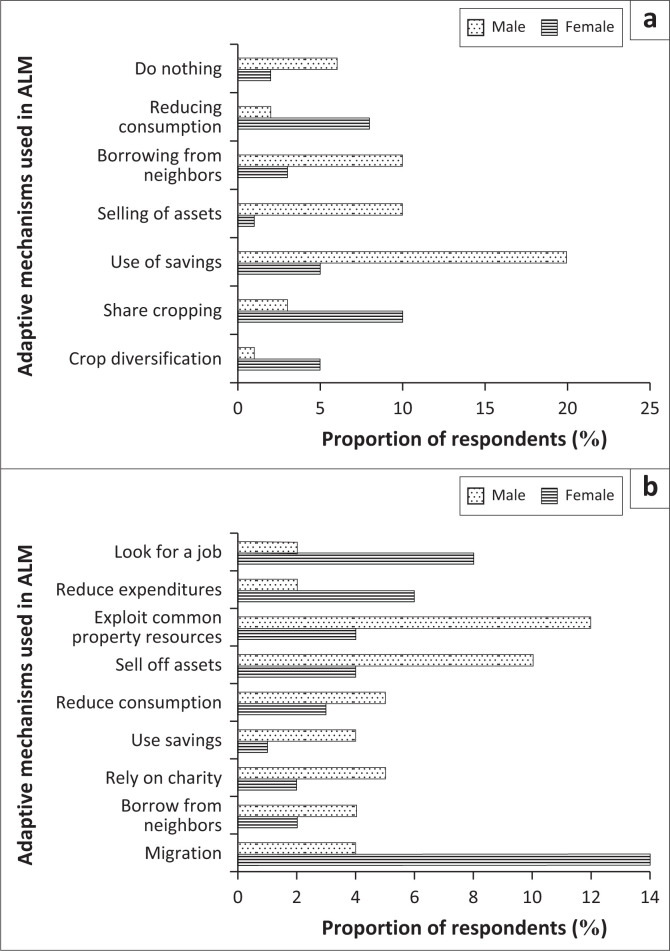
The different adaptive mechanisms adopted by the general population. (a) *during* a drought; and (b) *post*-drought in the Abaqulusi Local Municipality (ALM), KwaZulu-Natal Province, South Africa.
